# Genome Assembly of a Highly Aldehyde-Resistant Saccharomyces cerevisiae SA1-Derived Industrial Strain

**DOI:** 10.1128/MRA.00071-19

**Published:** 2019-03-28

**Authors:** Sheila Tiemi Nagamatsu, Gleidson Silva Teixeira, Fellipe da Silveira Bezerra de Mello, Pedro Augusto Galvão Tizei, Bruna Tatsue Grichoswski Nakagawa, Lucas Miguel de Carvalho, Gonçalo Amarante Guimarães Pereira, Marcelo Falsarella Carazzolle

**Affiliations:** a Departamento de Genética, Evolução, Microbiologia e Imunologia, Instituto de Biologia, Universidade Estadual de Campinas, Campinas, SP, Brazil; b Departamento de Engenharia de Alimentos, Faculdade de Engenharia de Alimentos, Universidade Estadual de Campinas, Campinas, SP, Brazil; Broad Institute of MIT and Harvard University

## Abstract

Here, we report the genome assembly of a Saccharomyces cerevisiae SA1-derived haploid (FMY097) indigenous strain isolated from a Brazilian ethanol distillery. FMY097 was recently reported to be a highly aldehyde-resistant strain capable of producing bioethanol in the presence of up to 40 mM furfural and 80 mM 5-hydroxymethylfurfural.

## ANNOUNCEMENT

The awareness of the environmental consequences resulting from fossil fuel usage leads to an increased interest in the development of renewable energy sources (1). In a biomass-derived ethanol context, different by-products with inhibitory effects on microorganism metabolism are significantly produced during industrial biomass pretreatment (2). In particular, aldehydes—such as furfural and 5-hydroxymethylfurfural (HMF)—induce cell wall and membrane damage and breakdown of DNA and inhibit protein and RNA synthesis (3). To overcome these effects, Saccharomyces cerevisiae strains adapted to industrial conditions (industrial strains) have been considered more appropriate platforms for the development of commercial yeasts able to convert C6 and C5 hydrolyzed sugars into ethanol (4, 5). Here, we report the genome assembly of a SA1-derived haploid (FMY097) indigenous strain isolated from a Brazilian ethanol distillery (6). FMY097 was recently reported to be a highly aldehyde-resistant strain, despite its high fermentation efficiency and prolonged persistence in the process, because it is able to produce biomass in the presence of up to 40 mM furfural and 80 mM HMF (7).

Genomic DNA was isolated with a Wizard genomic DNA purification kit (Promega) from haploid cells cultivated on yeast extract-peptone-dextrose (YPD) medium (1% yeast extract, 2% peptone, and 2% glucose). Illumina sequencing libraries were prepared using the Nextera DNA library prep kit and sequenced on a HiSeq 2500 instrument to produce around 8 million paired-end 100-bp reads, representing 134× genome coverage. The quality of sequenced reads was evaluated by the FastQC package (version 3) (8). The reads were assembled with SPAdes (version 3.6.1) (9) configured as the “–careful” parameter in order to decrease erroneous mutations and indels (insertions/deletions) and a k-mer ranging from 21 to 91 with a step of 10. The final assembly generated 228 contigs larger than 1,000 bp that totaled 11.6 Mb, an *N*_50_ value of 130,414 bp (28 contigs), and a G+C content of 36.8%.

Gene prediction was performed using AUGUSTUS software (version 3.2.3) (10) with default parameters and using the previously available “saccharomyces_cerevisiae_rm11-1a_1” as a training model. A total of 5,330 predicted proteins larger than 50 amino acids were identified, with an average sequence length of 508 amino acids. In order to evaluate the completeness of the predicted proteins, the BUSCO analysis (11) was applied using a set of 1,711 highly conserved orthologous Saccharomycetales proteins. A total of 99.5% of the 1,711 proteins in the BUSCO database were identified in the predicted proteins, and of these, 97.5% were classified as complete and single copy.

The annotation of predicted genes was performed using the well-studied genome of Saccharomyces cerevisiae S288c through the analysis of single-copy orthologs using the ORTHOMCL program (version 1.4) (12). A total of 4,747 single-copy genes were identified and annotated by this approach. The remaining 583 genes were annotated by PANNZER2 (version 2.0) (13), which resulted in only 51 unknown genes.

### Data availability.

This whole-genome shotgun project has been deposited at DDBJ/EMBL/GenBank under the accession number SDIA00000000 and BioProject number PRJNA515487. The DNA Illumina reads were submitted to the Sequence Read Archive (SRA) at NCBI under SRA accession number SRR8455574.
